# A Case of Acquired Factor V Deficiency in Patient with Bleeding

**DOI:** 10.1055/s-0039-3402024

**Published:** 2020-04-20

**Authors:** Davide Vetri, Giovanni Lumera, Salvatore Tarascio, Salvatore Scuto, Elisa Marino, Giuliana Barcellona, Salvatore Santo Signorelli

**Affiliations:** 1 Department of Clinical and Experimental Medicine, University of Catania, Catania, Sicily, Italy

**Keywords:** coagulation, acquired inhibitors, factor V

## Abstract

Low frequency of rare diseases origins from missed diagnosis addressing to poor prognosis. Acquired factor V inhibitor is a very low frequent bleeding condition (prevalence: 0.09/100,000,000–0.29/1,000,000 per year). Antibiotics, surgery, bacterial infections, malignancies, and autoimmune diseases are predisposing factors; however, no predisposing factor was found often. Authors reported a case of bleeding originating from an acquired factor V deficiency because they wish to draw attention to unfrequented or rare clinical situations rarely diagnosed in internal medicine unit.

## Introduction

Factor V (FV) plays a role in clotting. 1 FV deficiency is rare among bleeding disorders although it can ensue from an inhibitor antibody. Acquired factor V inhibitor (AFVI) prevalence is very low (0.09/100,000,000–0.29/1,000,000 per year) but its prevalence could be underestimated due to the absence of symptoms or missed diagnosis. 2 3 Predisposing factors are antibiotics (β-lactam, aminoglycoside, fluoroquinolone), surgery, bacterial infection, malignancies, and autoimmune diseases. The limited utilization of topical bovine thrombin has reduced its role in causing AVFI to arise. No predisposing factor was found in a considerable number of cases.

In a review, patients affected by AFVI were over 65 years (approximately 69 years) with men having a higher incidence (52 cases) than women (26 cases). 3 As for AFVI symptoms, bleeding from gastric, urinary, and respiratory mucosa was found most frequently (81%). A large percentage of AVFI patients (50%) showed hematuria. Postsurgery bleeding (16%) and hematoma (11%) are additional symptoms in AVF patients. Less frequent symptoms are intracranial (8%) and retroperitoneal bleeding (5%).

## Case Report

A 68-year-old woman with type-II diabetes, arterial hypertension (treated with a calcium antagonist), and chronic atrial fibrillation, was treated with amiodarone and direct oral anticoagulant (DOAC) therapy factor II inhibitor (dabigatran) from September 2016 to October 2018. At this time the patient showed bleeding (hematuria) and hematomas in the lower limbs and gluteus, so drug administration was stopped.

We screened for coagulation factors:

- Vitamin K-dependent coagulative factors (II, VII, IX, X) to evaluate vitamin deficiency or liver disease.
- No vitamin K-dependent coagulative factors VIII and V to exclude vitamin-K deficiency or acquired hemophilia A. 4

We dosed markers of viral liver infection to exclude liver disease as alternative diagnosis of consumptive coagulopathy. Lupus anticoagulant, anticardiolipin autoantibodies, anti β2-microglobulin were dosed to diagnose an autoimmune disease.

We found a very low level of FV (0.1% vs. normal value 60–140%)—no other coagulative factors were altered ( Table 1 ). Autoantibody research did not prove positive. We applied the Mixing test by measuring coagulation time, i.e., international normalized ratio (INR), activated partial thromboplastin time (aPTT) ratio at time 0 and 2 hours after incubation at 37°C. Correction in coagulation time after the Mixing test was not found. Based on these findings, we postulated that the low FV level ensued from AFVI (measured as 1.94 BU on the Bethesda units scale).

**Table 1:** Patient’s laboratory values of coagulative factors

Coagulative factor	Lab value (%)	Range
Factor II	68.9	n.v. 50–150
Factor V	0.1	n.v. 50–150
Factor VII	68.4	n.v. 50–130
Factor VIII	115	n.v. 50–150
Factor IX	141	n.v. 65–150
Factor X	70.5	n.v. 50–150
Factor XI	91.9	n.v. 65–150
Factor XII	72.1	n.v. 50–150

## Therapy

**Fig. 1:**
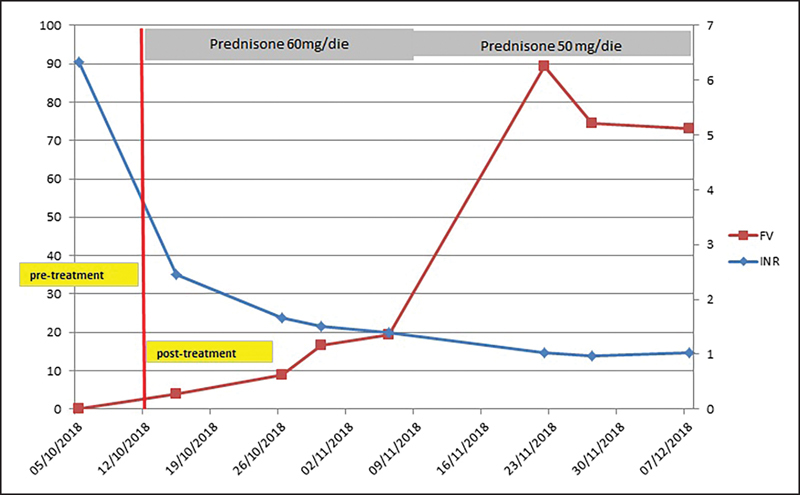
Trend of values of INR and FV before and after immunosuppressive treatment. FV, factor V; INR, international normalized ratio.

Patient was prescribed the following drugs: prednisone (1 mg/kg, 60 mg/daily) and cyclophosphamide (100 mg/daily). Antihemorrhage therapy was administrated by using concentrated activated prothrombin complex, human plasma factor VIII inhibitor, and bypassing activity (FEIBA; 70 UI/kg). We reduced FEIBA dosage on the seventh day and suspended it on the twelfth day. The cyclophosphamide dose was reduced to 50 mg/daily for 45 days. Prednisone was progressively lowered to a 25-mg daily dose. The diagnostic procedure to find the cause of AFVI went on: both antibiotics and surgery were excluded, negative results were found for antinuclear antibodies, complement systems (C3, C4), and extractable nuclear antibody screening. Therefore, we could exclude an autoimmune pathogenesis of AFVI. CT scans of chest, abdomen, and pelvis were negative for malignancies. Based on the above results, we postulated that bleeding was provoked by idiopathic AFVI. Bleeding (hematuria) ceased 5 days after the beginning of the treatment, hematomas progressively disappeared. Patient follow-up (complete blood count, INR, aPTT, and FV) went on for 2 months ( Fig. 1 ).

## Discussion

Several diagnoses may be postulated for patients affected by bleeding (i.e., platelet deficiency including disseminated intravascular coagulation [DIC], idiopathic thrombocytopenic purpura [ITP], and thrombotic thrombocytopenic purpura [TTP]). Alternatively, bleeding can originate from the deficiency of coagulative factors linked to vitamin-K deficiency, liver disease, or caused by acquired inhibitors. Acquired inhibitor factor is a rare cause of bleeding, which may be due to an autoantibody against factor VIII (1.3–1.5 cases per million population per year) or factor II (most often detected in patients with antiphospholipid antibodies) or, less frequently, factor V. So, autoantibody against the factor VIII is the most frequent diagnosis among patients with bleeding caused by an acquired inhibitor factor. 1 We exclude the diagnosis of FVIII inhibitor because FVIII plasmatic level was normal (115%, n.v. 50–150) and it was confirmed by Mixing test result. Family history of coagulative deficiencies, drugs, and previous diseases, along with unusual blood tests can be helpful in diagnosing acquired inhibitor factors. Our patient had undergone DOAC therapy only 2 years before, and the drug was suspended a short time before hospitalization. The duration of the DOAC therapy and the persistence of bleeding despite DOAC suspension (10 days before hospitalization) are robust evidence to exclude interaction between DOAC and AFVI. On the other hand, both the normal platelet count and high fibrinogen plasma level ( Table 2 ) are the keys to exclude a consumption coagulopathy (i.e., DIC, ITP). Regular liver activity, negative markers for liver infection, no pathological ultrasound findings, and negative CT scan were helpful to exclude liver disease. The Mixing test was fundamental in diagnosing AFVI, because it showed factor V inhibitor and its plasma concentration measured in Bethesda units scale. It is known that FEIBA includes vitamin K-dependent factors (II, VII, IX, X factors), so no FV replacement was administered. Despite this, bleeding (hematuria) ceased after 5 days and INR quickly normalized. Immunosuppressant treatment improved the plasma level of FV. There was no collateral effect by immunosuppressant drugs (i.e., hemorrhagic cystitis, worsening hematuria).

**Table 2:** Patient’s coagulative and metabolic data and their normal range

Patient’s lab data	Lab value	Range
Hemoglobin	4.1 a	11.7–16 g/dL
Hematocrit	13.2 a	36–48%
MCV	89	80–99 fL
Platelets	237	150–400 × 10 ^3^ /µL
Leukocytes	9.32	5.20–12.40 × 10 ^3^ /µL
Neutrophils %	77.4 a	40–74%
Lymphocytes %	15.4 a	19–48%
Monocytes %	6.4	3.4–9%
Eosinophils %	0.5	0.5–7%
Basophils %	0.3	0–1.5%
PTT%	11 a	70–130%
INR	6.48 a	0.8–1.2
Fibrinogen	589 a	170–450 mg/dL
D-dimer	405 a	0–250 µg/L
Creatinine	0.94	0.51–0.95 mg/dL
AST	32	0–35 U/L
ALT	20	0–35 U/L
GGT	18	0–38 U/L
LDH	185	0–248 U/L
Na	140	135–145 mmol/L
K	4	3.5–5 mmol/L
Cl	106	101–109 mmol/L
Mg	2	1.9–2.5 mg/dL
CRP	21.2 a	0–10 mg/L

Abbreviations: ALT, alanine aminotransferase; AST, aspartate transaminase; CRP, C reactive protein; INR, international normalized ratio; GGT, gamma glutamil transaminase; LDH, lactate dehydrogenase; MCV, mean cell volume; PTT, prothrombin partial time.

a Lab tests helpful to diagnose bleeding occurrence.

For patients affected by bleeding but not diagnosed for common coagulative diseases, it is worth looking at less frequent diagnoses including AFVI. We hope that this case report can be useful because AFVI diagnoses are uncommon in clinical practice. Based on work by our internal medicine unit, AFVI is a rare diagnosis compared with other hemorrhagic diseases most frequently diagnosed in patients hospitalized for bleeding. 5 6

We hope these details are useful to raise awareness about an unusual coagulative factor deficiency in patients with active bleeding.
